# ADVANCED CARE PLANNING SERVICES BILLING ENCOUNTERS AMONG MEDICARE BENEFICIARIES WHO DIED IN 2019

**DOI:** 10.1093/geroni/igad104.3165

**Published:** 2023-12-21

**Authors:** Fred Kobylarz, Hyosin Kim, Anum Zafar, Haiqun Lin, Maria Lopez, Olga Jarrín

**Affiliations:** Rutgers Robert Wood Johnson Medical School, New Brunswick, New Jersey, United States; Oregon State University, Corvallis, Oregon, United States; Rutgers, The State University of New Jersey, New Brunswick, New Jersey, United States; Rutgers, The State University of New Jersey, Newark, New Jersey, United States; Rutgers, The State University of New Jersey, New Brunswick, New Jersey, United States; Rutgers, The State University of New Jersey, New Brunswick, New Jersey, United States

## Abstract

In 2016, the Centers for Medicare & Medicaid Services (CMS) introduced advanced care planning (ACP) services Current Procedural Terminology [CPT] codes 99497 (first 30 minutes) and 99498 (additional 30 minutes). This enabled healthcare providers to seek reimbursement for discussing future medical care preferences when patients are unable to make decisions. In this study, we linked administrative, claims, and encounter files for the 2,238,611 Medicare beneficiaries who died in 2019. Of these, 17.7% (395,258) had a billing code for ACP services in 2018 or 2019. Among fee-for-service (FFS) beneficiaries, 18.8% had ACP services billed compared to 15.7% of Medicare Advantage (MA) beneficiaries. Most ACP encounters were for the first 30 minutes of services (91% FFS, 90% MA). Among FFS beneficiaries, most ACP encounters were attributed to internal medicine (30.8%), family medicine (12.6%), and nurse practitioners (29.9%); and took place in hospitals (47.6%), skilled nursing facilities (18.4%), and office (14.2%) settings. Location of ACP services was available for only 8% of MA encounters: hospitals (40.3%), skilled nursing facilities (18.2%), and office (20.1%). Additional findings will be presented regarding variation in ACP encounters by race/ethnicity and geographic region (state, rural/urban community, area deprivation index) that enhance our understanding of ACP service use at the end-of-life for Medicare fee-for-service and Medicare Advantage beneficiaries.

